# The Pattern of Rare Earth Elements Like a Possible Helpful Tool in Traceability and Geographical Characterization of the Soil-Olive System (*Olea europaea* L.)

**DOI:** 10.3390/plants11192579

**Published:** 2022-09-30

**Authors:** Marcella Barbera, Filippo Saiano, Livia Tutone, Roberto Massenti, Antonino Pisciotta

**Affiliations:** 1 Dipartimento Scienze Agrarie, Alimentari e Forestali, Università degli Studi di Palermo, Viale delle Scienze Ed.4, 90128 Palermo, Italy; 2 Dipartimento Géosciences, Ressources Naturelles et Environnement, Sorbonne Université, METIS, 4 place Jussieu, 75005 Paris, France

**Keywords:** rare earth elements, normalized pattern, icp-ms, olive, traceability

## Abstract

The identification of a product, with its geographical origin, is a guaranty of the value of the foodstuff and protection from potential fraud. Extra virgin olive oil is produced or marketed as a single variety or a blend of two or more cultivars, often of different geographic origins. Therefore, to study a possible link between the soil and olive oil, we accounted crucial to analyse the behaviour of olive of different cultivars. We studied Rare Earth Elements (REE) amounts and their relationship to trace their distribution from soil to the olive pulp (*Olea europea L.*). The results obtained pointed out that the different cultivars of Olea did not drive significant differences in reciprocal ratios of REE in the uptake from the soil up to olive (except for Eu). However soil-plant Rare Earth relationships depend exclusively on the soil REE composition. This method can be the starting point to enforcing the laws, in fact, it is important to develop analytical methods to measure the authenticity of the samples, and to verify the geographical origin even when olive oil is blended.

## 1. Introduction

The possibility of tracing the origin of foodstuff is assuming an increasingly important role as a tool that may allow product authenticity to be proven and adulteration to be controlled [1]. The rising demand for olive oils of high quality from consumers, the importance attributed by legislators to guaranteeing the provenance of products from the olive sector and the health and safety of consumers, has motivated the identification of the geographical traceability of agriculture products, especially the need for a reliable monitoring system in the food sector [2,3,4,5,6,7,8,9,10]. In this field, extra virgin olive oil (EVOO) is considered one of the most relevant Italian agricultural commodities. Several authors reported that the health effects of the Mediterranean diet can be related to the consumption of extra virgin olive oil due to the high content of antioxidant substances [11,12,13]. However, EVOO superior quality and commercial value derive widely from the geographical origin of production and the varieties. The designation recognition supposes the investigation of several processes along the entire EVOO production chain: olive cultivation, oil extraction and bottling, with particular attention to the cultivars used for the production of a specific oil, the geographical area of cultivation, followed by management practices, production/extraction and organoleptic attributes. The analytical methods commonly applied must use adequate standard and appropriate statistics [14,15,16,17]. The improvement of the detection limits of several instruments has allowed the development of analytical methods based on Rare Earth Elements (REE: Lanthanum (La), Cerium (Ce), Praseodymium (Pr), Neodymium (Nd), Samarium (Sm), Europium (Eu), Gadolinium (Gd), Terbium (Tb), Dysprosium (Dy), Holmium (Ho), Erbium (Er), Thulium (Tm), Ytterbium (Yb) and Lutetium (Lu)) as food markers [6,7,8,18,19,20,21]. These elements are relatively abundant in the Earth’s crust, and Yttrium (Y), due to its chemical similarity, is generally studied with them. Geographical or variety characterization cannot be documented by chemical compounds that are not stable during the olive oils’ life [22] and REE are very important because their concentration does not change over time, as they could be representative of the soil. A study has shown that REE are tracers of soil and/or phosphorus fertilizers in use [23] and, in another study, they were used for the identification of the geographical origin of pumpkin seed oil [24].

The characteristic behaviour of REE has allowed elucidation of the mechanisms of their uptake by plants, representing a helpful tool to analyze plant-soil system transfer processes [2,25,26,27,28]. These elements are directly linked to the geology of the area; therefore, their profile reflects the geographical origin of the cultivation area. It is known that REE in plants have a characteristic distribution pattern similar to geological samples [29]. Their local differentiation, through mobilization and redistribution processes, could be represented by the ∑HREE/∑LREE ratio (HREE Heavy REE from Terbium to Lutetium plus Yttrium, LREE Light REE from Lanthanum to Gadolinium) and by the so-called normalized patterns, and together, in this more geochemical approach, seem to be a promising tool to establish univocal traceability systems in the agro-food field [30,31,32]. It is significant to highlight that REE multi-element distribution can be a helpful tool for the determination of the geographical origin of food products if the REE distribution is preserved from the soil to the plant irrespective of the mechanisms involved in metal uptake and transfer by plants. Analogously to how we have made on *Vitis vinifera*, an essentially aqueous system, we have tested the same REE geochemical approach, precisely to verify its ability, also in a widely different apparatus as the olives, a very low-aqueous system.

For all these reasons, and because it is also known that the produced or marketed EVOO is frequently a blend of olives of two or more cultivars and/or of the same cultivar but from different geographical origins, we have decided to start the study of the soil-olive fruit link as the first step of the EVOO production chain. Therefore, in this paper, we initially studied the distribution of REE between a single soil (a single soil by point of view of the REE distribution) and 38 indigenous Sicilian olive cultivars to establish if the distribution pattern remains unaltered irrespective of the characteristics of each variety.

## 2. Materials and Methods

### 2.1. Chemicals

Concentrated nitric acid (65%) and hydrogen peroxide (30%) of ultrapure grade were purchased from Baker (Milano, Italy), diethylenetriaminepentaacetic acid (DTPA) (purity > 99%) was from Sigma-Aldrich (Italy). Ultrapure water 18.2 MΩ cm, produced with an EASYpureII (Thermo, Milano, Italy), was used for all standard solutions and sample preparations. Y, Lanthanoids, Rh (Rhodium) and Re (Rhenium) standard solutions (1000 ± 5 g mL^−1^) were purchased from BDH, Merck and CPI International (Milan, Italy).

### 2.2. Experimental Layout, Plant Material, and Sampling

Soil and olive (*Olea europaea* L.) samples were provided by the Experimental Station of the Agricultural Development Agency (ESA) of the Sicilian regional government located in Castelvetrano (Sicily, Italy). The Experimental field “Campo Carboj” (37°34′59.20″ N, 12°52′32.12″ E) covers 16.36 hectares in a flat area (50 m a.s.l.) with a silty soil texture. Rainfall amounts are around 500 mm/year (Sicilian Agrometeorological Information Service, SIAS source, http://www.sias.regione.sicilia.it/ accessed on 11 September 2022), concentrated during autumn-winter. Although during the winter season the minimum temperature rarely falls below 3–4 °C, in contrast, during the summer season, the maximum temperature frequently exceeds 40 °C. The sampled olive trees were 8 years old, trained to vase system, spaced at 5 m × 7 m (285 trees ha^−1^) and drip irrigated. Soil management practices included resident vegetation during winter and incorporating the biomass into the soil in April by ploughing.

Three to four shallow tillages (10–12 cm deep), from spring to summer, were carried out to control weeds and prevent soil cracking [33]. Thirty-eight indigenous Sicilian olive cultivars (replicated three times with 3 plants), were used from the germplasm collection of Campo Carboj (Table 1). At harvest (autumn-winter), a representative sample (replicated three times) of 1 kg of olive was randomly collected from nine plants of each cultivar and representative soil samples (about 2 kg), were also collected in the same plot where the olives were harvested (depth from 10 to 40 cm, distance from the tree 1.5 m).

### 2.3. Sample Preparation

All soil samples were treated as reported in [32]. To extract the REE pseudo-total fraction (which contains all the elements without those blocked in silicates) aliquots of 0.5 g (dried weight) were digested using HNO_3_ and H_2_O_2_ in a microwave system (Supplementary Material Table S1) while to obtain the REE bioavailable fraction, aliquots of 10.0 g were treated with 20.0 mL of a (DTPA) 0.005 M (at pH 5.0 with NaOH) and shaken for 24 h at room temperature. Each analytical sequence included a procedural blank (ultrapure water treated as the other samples).

The olive samples were washed with ultrapure water, and the pulp separated from the pit. To pulp aliquots (2.0 g dried weight) in open vessels, 4 mL of HNO_3_ of ultrapure grade were slowly added two times, spaced one half-hour apart to avoid the tumultuous formation of gas and sample leaks, and digested in a microwave system (Mars 5 Xpress, CEM, Italy). In a second mineralization step, the samples, to which 2.5 ml of ultrapure-grade H_2_O_2_ were added, were newly digested (Supplementary Material Table S1). After digestion, the extracts were diluted with ultrapure water to 15.0 mL. Ultrapure water, treated as a sample, was used as a procedural blank.

### 2.4. Elemental Analysis

ICP-MS measurements (Agilent Technologies 7500ce Series Spectrometer, Milano, Italy) were carried out as in [32]. In particular, ICP-MS was carefully tuned by monitoring ^7^Li, ^59^Co, ^89^Y, ^140^Ce and ^205^Tl masses and optimised, with a solution containing 1.0 μg mL^−1^ of Ba in a 3/1 *v/v* ratio of HNO_3_/H_2_O_2_, to evaluate both ratios ^135^Ba^16^O^+^/^135^Ba^+^ and ^134^Ba^17^O^+^/^134^Ba^+^, the higher amount of which was below 0.1%, to better control the Ba interferences on the determination of ^151^Eu. The REE recovery in olive pulp was estimated by analysing the INCT-OBTL-5 Oriental Basma Tobacco Leaves certified standard material [34]. Values, between 95% and 105%, were found for certified elements (Lanthanum, Cerium, Neodymium, Samarium, Europium, Terbium, Erbium and Ytterbium) and from 98% to 110% for elements whose values were listed as informative. Lutetium showed a low value of 75% (Supplementary Material Table S2). In the soil, the quality of pseudo-total REE content of our samples was verified by analysing the SRM 2586 (trace elements in soil containing lead from paint) from the National Institute of Standards and Technology (NIST). The REE recoveries showed an acceptable agreement between certified and observed concentration values (Supplementary Material, Table S2). LOQ was estimated by using the following equation YQL = y + 10s where y is the average value of the standard solution signal and s is the standard deviation obtained by measuring at least ten different aliquots of standard solution at REE concentration near the lower point of the working range (1 ng/L). Data obtained for each element are shown in Supplementary Material Table S2.

### 2.5. Data Analysis

The software SYSTAT (ver.10; Systat Software Inc., San Jose, CA) was used for statistical analyses. The cultivar was considered the main factor, and one-way analysis of variance (ANOVA) was performed (α = 0.05). Statistically significant differences between means were determined using Tukey’s HSD test (α = 0.05).

The distribution coefficient values (K_d_), were calculated as [REE]_1_/[REE]_2_, between REE concentrations measured in two interfaced substances (for example, 1 = olive pulp and 2 = soil).

## 3. Results and Discussion

### 3.1. Soil

The REE availability for plants is typically controlled by the solubility and stability of REE complexes in the rhizosphere [35], and for this reason, their determination in bedrock is not as useful as the bioavailable and pseudo-total fractions in soil [32]. Our averaged concentration data are reported in Supplementary Material Table S3.

We analysed the REE amount as normalized data, i.e., by the ratio between the molar concentration of each sample to the molar concentration of one of the several international references reported in the literature, in our case, to the Upper Continental Crust (UCC) [36].

**Figure 1:**
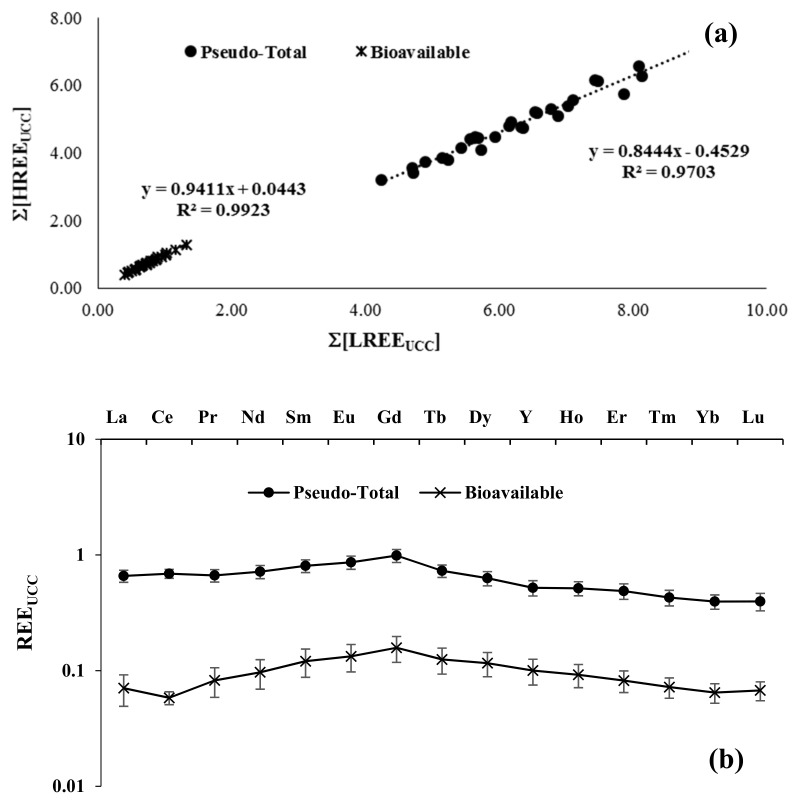
(**a**) ∑[HREE_UCC_] vs. ∑[LREE_UCC_] relation for all Campo Carboj soil samples considering both treatments (pseudo-total and bioavailable). The scales are logarithmic. (**b**) UCC-normalised REE patterns of Campo Carboj soils. Averaged values with standard deviation for both pseudo total and bioavailability treatments.

Figure 1a reports the ∑[HREE_UCC_] vs. ∑[LREE_UCC_] relation for all soil samples, obtained considering both methods (pseudo-total and bioavailability). The linearity of this relation, with a very good R^2^ (0.955 and 0.991, respectively), confirms the homogeneity of the REE distribution. The different slope of samples obtained with the system of bioavailability is due to the different complexation constants of HREE and LREE with diethylenetriaminopentaacetic acid (DTPA) [37]. However, in REE studies, as reported by [38], the relative distributions of concentrations give more information than single absolute concentration values.

As we underlined in the introduction, considering the REE as a homogeneous group, their normalized concentration ratios, analogously for example to ^87^Sr/^86^Sr isotopic ratio, are more useful than absolute values when we consider the geochemical approach: soils normalized pattern with identical shape, but with different absolute amounts, therefore parallel patterns, geochemically represent the same soils for the REE tracers. The soil samples normalized averaged patterns, both for pseudo-total and bioavailable treatments (Figure 1b), showed very low standard deviation values and reflected very good REE markers spatial homogeneity in the studied area. Therefore, the Campo Carboj, regarding the REE distribution, appears as an overall unique soil in which different olive trees grow. A flat REE distribution with a progressive slight decrease from Gd until Lu is evident in the pseudo-total soil fractions. No significant anomaly in REE distribution is present. Particularly, Ce and Eu, concerning their specific chemical behaviour (two oxidation states for Ce and the similar behaviour of Eu with Ca), were the principal elements that could show fractionation. On the other side, a slight increase from La to Gd with a small negative anomaly of Ce is observed in the REE pattern of the bioavailable soil fraction. The behaviour from Gd to Lu is superimposable on pseudo-total fractions. The oxidant and more acidic (pH < 1) strength of the pseudo-total treatment justify the higher REE amount released from soil samples in comparison with DTPA treatment (pH =5). The different DTPA-REE complexation constant values cause the behaviour of LREE to respect HREE and the Ce anomaly [37].

### 3.2. Olive Pulp

**Figure 2:**
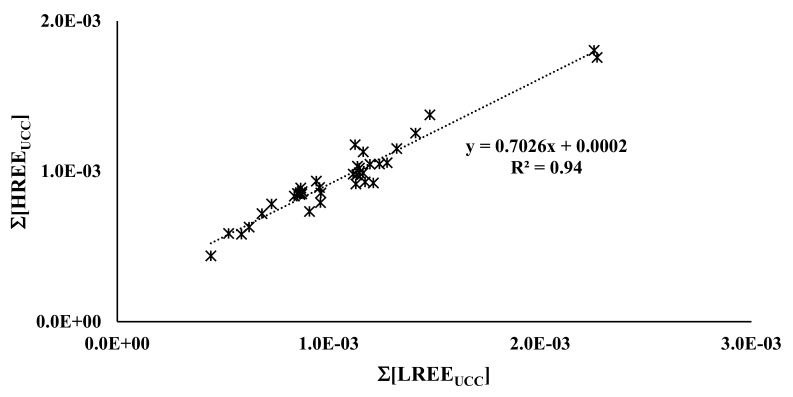
∑[HREE_UCC_] vs. ∑[LREE_UCC_] relation for all Campo Carboj olive pulp samples.

**Figure 3:**
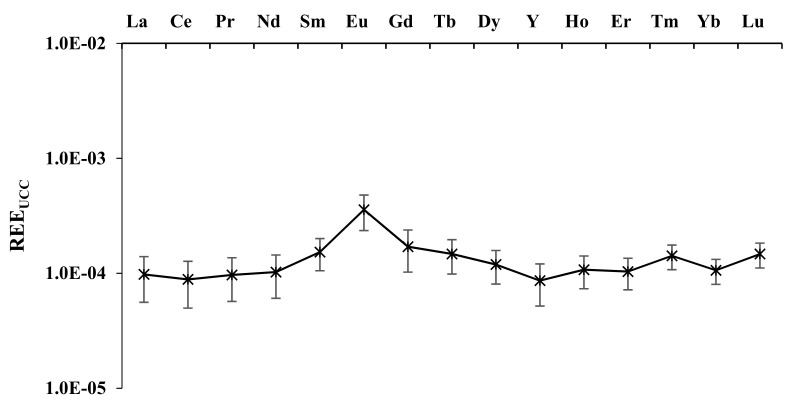
UCC-normalised REE pattern of Campo Carboj olive samples (averaged values with standard deviation for all samples).

Very scarce are the data about trace elements and, in particular, REE distributions in *Olea europaea * L. However, as in other food products, the behaviour seems typical with a low amount in olive pulp [3,39,40]. Our results of REE concentrations, from olive pulp analysis for each cultivar, are reported as average values with standard deviations, in nanomoles kg^−1^ in Table 2. Before applying the “geochemical” treatment used with soil, we initially carried out a simple statistical approach. The statistical analysis (ANOVA), working with REE absolute amounts, supported a significant effect of cultivars on the uptake of all REE elements. Particularly, we found the strongest uptake from *CVs* “Passulunara”, “Minuta”, and “Bottone di Gallo”. However, as previously reported [32], the climatological conditions and physiology of different olive trees (the differences in expression of vigour), reasonably, seem to influence only the total amount of REE uptake and not their reciprocal ratio. Therefore, ∑[HREE_UCC_] vs. ∑[LREE_UCC_] and the UCC normalized patterns of the olive pulp for each different cultivar were evaluated. Figure 2 shows the ∑[HREE_UCC_] vs. ∑[LREE_UCC_] relation for all olive pulp samples. Very intriguing results were obtained: a high R^2^ value, 0.94, independently by any cultivar considered or its peculiar agronomic features. The meaning is that all plants showed the same correlation. Analogously, the REE pattern of the averaged concentration values of all the different cultivar olive samples, shown in Figure 3, is very significant. In fact, within the values of the standard deviations, the differences between the different samples are very small, confirming that every cultivar has similar behaviour in the uptake of REE. The noteworthy fractionation of Eu is present, a common feature in REE plant uptake, as previously reported in studies of the uptake of REE by grapes [30,32]. Eu seems to be preferentially mobilised from soil to olive pulp, as it is known, for its great similarity concerning Ca [27,35].

### 3.3. Plant/Soil Relationships

**Figure 4:**
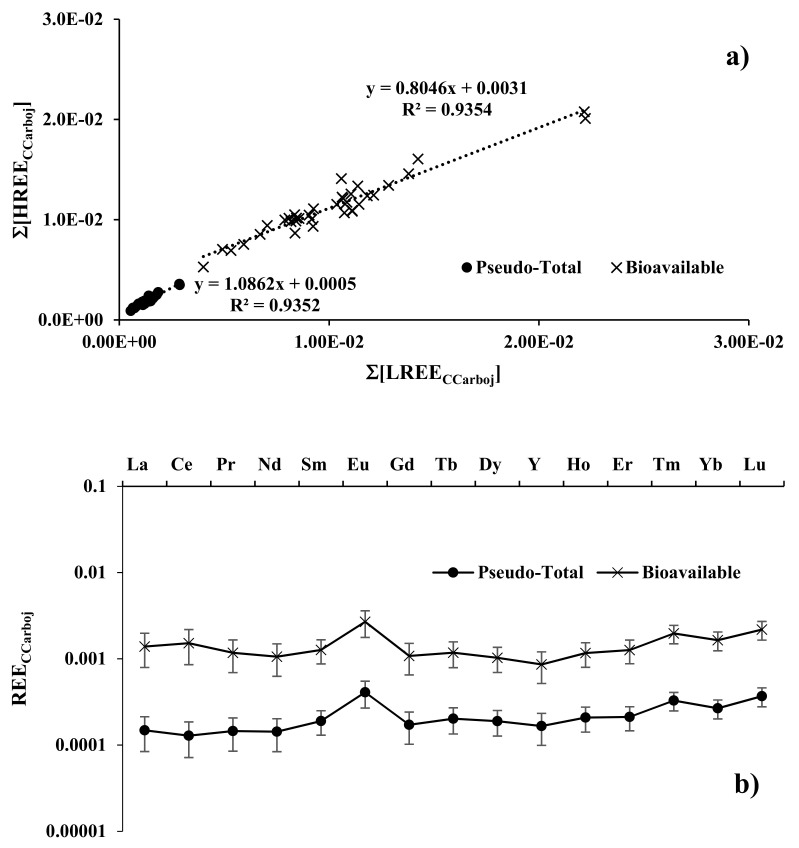
(**a**) ∑[HREE_CCarboj_] vs. ∑[LREE_CCarboj_] relation for all olive pulp samples. (**b**) Pseudo-total and Bioavailability Campo Carboj-normalised REE patterns (K_d_) of investigated olive oil samples.

In the olive tree/soil system, to identify a direct correlation between REE contents in olive pulp and the amount determined in its soil, we calculated K_d_ values. We found that a linear correlation, in the ratio ∑[HREE]_CCarboj_ vs. ∑[LREE]_CCarboj_ for both normalizations (Figure 4a), is strongly maintained (R^2^ 0.935 for both the methods) meaning that olive pulp from different cultivars grown in the same soil presented the same UCC-normalized REE pattern. The K_d_ coefficients, both for pseudo-total and bioavailable fractions (Figure 4b) were always independent of the cultivar and, as expected, all characterised by a positive Eu anomaly. Along with the REE series, principally flat features (with Eu exception) in both soil fractions were present. The slight Ce-positive anomaly in the olive pulp for DTPA soil fraction is a consequence of the distribution coefficient K_Ce_ where the denominator is lower for the Ce-negative anomaly present in the DTPA soil fraction. Similar considerations can be made for the behaviour of Eu.

## 4. Conclusions

The overall results of this preliminary study with this approach, to our knowledge, are the first on olive pulp. The obtained data, and the pattern among different varieties, show that the ratios and distributions of REE of this “homogeneous” soil are not influenced (with the expected exceptions of Eu) by different *Olea europaea L.* cultivars: indeed, all the olives of different cultivars maintain the soil fingerprint. These encourage further research on the ability of the ∑[HREE]_UCC_ vs. ∑[LREE]_UCC_ relation, REE normalized patterns and K_d_ factors to link more directly to the olive-soil system. In this geochemical approach applied, the normalising factors are useful in evidencing any possible similarity or difference in the samples of the studied systems. In perspective, this easy and relatively expensive methodology, once the REE features (∑[HREE]_UCC_ vs. ∑[LREE]_UCC_ ratio and normalized patterns) have been characterised for more and more soils of other countries, could be applied both in table olive and olive oil and, more generally, to other agro-food products, particularly, in terms of geographical traceability as well in adulteration control.

## Figures and Tables

**Table 1:** The indigenous Sicilian olive cultivars studied.

N	Cultivar
1	Pizzutella
2	Nuciddara
3	Verdella
4	Rotondella
5	Selvatico
6	Ogliara
7	Tonda Iblea 1
8	Zaituna
9	Nocellara del Belice
10	Minuta
11	Cerasuola 1
12	Pizzo di Corvo
13	Tonda Iblea 2
14	Passulunara
15	Moresca 1
16	Opera Pia Castelnuovo
17	Nocellara Belice
18	Olivo di Castiglione
19	Bariddara o Barilara
20	Moresca 2
21	Nocellara Belice SO
22	Iacona
23	Nocellara Messinese
24	Monaca
25	Cacaridduna
26	Cirasuola
27	Tonda Iblea 3
28	Ebano Ita
29	Cerasuola 2
30	Tunnulidda
31	Moresca 3
32	Vetrana
33	Fastucara (Aragona)
34	Lunga del Vassallo
35	Ebano
36	Cerasuola 3
37	Bottone di gallo
38	Passulunara (Naro)

**Table 2:** Concentrations of REE, from analysis for each olive pulp cultivar (Table 1), averaged values with standard deviations, in nmoles Kg^−1^ (** = *p* ≤ 0.01).

Cv	La		Ce		Pr		Nd		Sm		Eu		Gd		Tb		Dy		Y		Ho		Er		Tm		Yb		Lu	
	av	±σ	av	±σ	av	±σ	av	±σ	av	±σ	av	±σ	av	±σ	av	±σ	av	±σ	av	±σ	av	±σ	av	±σ	av	±σ	av	±σ	av	±σ
**1**	31.5	0.5	60.5	1.0	6.8	0.2	25.6	0.6	6.0	0.4	2.5	0.3	5.3	0.3	0.83	0.05	3.5	0.1	30.3	1.4	0.73	0.03	2.1	0.1	0.33	0.02	1.6	0.1	0.35	0.01
**2**	15.0	0.5	28.0	1.2	3.5	0.2	12.8	0.7	3.7	0.2	2.3	0.1	2.9	0.1	0.42	0.03	2.0	0.1	16.5	0.7	0.38	0.01	1.5	0.1	0.21	0.03	1.0	0.1	0.22	0.02
**3**	26.7	0.9	50.7	0.8	6.0	0.2	21.0	0.4	5.0	0.5	2.0	0.1	4.6	0.2	0.64	0.04	2.8	0.2	24.0	0.7	0.52	0.02	1.6	0.2	0.25	0.02	1.3	0.1	0.28	0.02
**4**	18.4	0.2	35.3	1.2	4.5	0.1	16.6	0.4	4.3	0.2	1.9	0.1	3.6	0.1	0.54	0.02	2.4	0.2	19.2	0.7	0.46	0.05	1.6	0.1	0.23	0.02	1.2	0.1	0.26	0.02
**5**	22.3	0.2	42.4	0.9	5.1	0.1	19.8	0.2	4.6	0.2	2.6	0.1	4.1	0.1	0.59	0.06	2.6	0.2	22.3	0.1	0.51	0.05	1.8	0.6	0.25	0.02	1.2	0.1	0.27	0.01
**6**	21.4	1.1	41.6	1.8	5.2	0.3	19.4	1.0	4.5	0.3	2.4	0.2	4.1	0.4	0.56	0.01	2.6	0.1	22.6	1.7	0.54	0.03	1.7	0.2	0.23	0.01	1.3	0.1	0.24	0.04
**7**	20.4	0.3	39.4	0.9	4.9	0.4	17.9	0.6	4.8	0.7	2.4	0.3	4.1	0.4	0.74	0.26	2.7	0.4	20.6	0.5	0.59	0.16	1.9	0.4	0.39	0.23	1.4	0.2	0.43	0.23
**8**	12.0	0.1	22.9	0.3	2.8	0.1	10.3	0.3	3.0	0.3	1.2	0.1	2.3	0.1	0.36	0.02	1.5	0.1	12.2	0.5	0.33	0.03	1.2	0.1	0.20	0.01	0.9	0.1	0.20	0.01
**9**	18.7	0.2	36.2	0.7	4.0	0.1	15.3	0.4	4.0	0.3	1.3	0.1	3.5	0.1	0.53	0.04	2.2	0.2	19.1	0.4	0.45	0.03	1.6	0.2	0.22	0.02	1.2	0.1	0.24	0.02
**10**	49.7	1.7	96.9	5.9	11.4	0.3	43.4	1.6	9.1	0.4	4.1	0.1	8.4	0.2	1.19	0.05	5.3	0.2	52.8	1.3	0.98	0.04	3.0	0.2	0.41	0.02	2.3	0.1	0.40	0.02
**11**	11.4	0.1	25.0	0.4	3.2	0.1	13.8	0.2	4.0	0.2	2.0	0.1	3.4	0.1	0.50	0.05	2.3	0.2	21.7	0.4	0.47	0.02	1.4	0.1	0.26	0.01	1.1	0.1	0.22	0.03
**12**	8.2	0.2	16.2	0.1	2.2	0.1	8.7	0.4	2.6	0.4	1.4	0.2	2.1	0.1	0.36	0.03	1.4	0.1	10.0	0.1	0.30	0.04	1.0	0.1	0.18	0.02	0.9	0.1	0.16	0.02
**13**	20.6	0.4	40.8	0.4	4.6	0.1	18.5	0.9	4.6	0.2	2.5	0.1	4.3	0.3	0.65	0.04	2.7	0.1	24.6	0.7	0.61	0.04	1.7	0.2	0.29	0.03	1.5	0.1	0.27	0.03
**14**	90.7	0.4	186.1	0.5	31.1	0.1	134.7	0.5	32.1	0.3	7.5	0.1	30.9	0.3	4.40	0.10	22.8	0.4	189.1	0.4	4.51	0.02	12.4	0.2	1.73	0.05	10.4	0.1	1.59	0.05
**15**	17.6	0.2	34.5	0.1	4.3	0.1	16.0	0.5	4.1	0.3	1.9	0.2	3.7	0.1	0.55	0.02	2.5	0.1	21.3	0.1	0.51	0.03	1.6	0.2	0.24	0.02	1.2	0.1	0.25	0.03
**16**	17.9	0.4	33.5	0.4	4.4	0.1	16.4	0.4	4.5	0.6	1.4	0.1	3.6	0.2	0.56	0.01	2.4	0.2	18.5	0.1	0.44	0.01	1.4	0.1	0.26	0.02	1.2	0.1	0.23	0.02
**17**	9.0	0.3	17.4	0.5	2.2	0.1	8.6	0.2	2.6	0.1	1.1	0.1	2.0	0.1	0.33	0.02	1.5	0.2	10.2	0.1	0.31	0.02	1.0	0.1	0.19	0.01	0.9	0.1	0.19	0.05
**18**	6.5	0.2	11.9	0.1	1.6	0.1	5.8	0.1	2.3	0.2	1.0	0.1	1.5	0.2	0.25	0.01	1.1	0.1	8.1	0.1	0.20	0.02	0.8	0.1	0.14	0.01	0.7	0.1	0.15	0.03
**19**	17.5	1.0	34.0	0.6	4.2	0.2	16.2	0.6	4.3	0.2	1.4	0.1	3.8	0.2	0.55	0.04	2.4	0.1	19.3	0.3	0.47	0.03	1.6	0.1	0.25	0.01	1.3	0.1	0.26	0.02
**20**	18.1	0.1	36.2	0.4	4.4	0.1	17.2	0.5	3.9	0.3	1.4	0.1	3.6	0.1	0.56	0.03	2.2	0.1	21.3	0.1	0.44	0.03	1.7	0.2	0.23	0.01	1.1	0.1	0.23	0.02
**21**	16.1	0.3	31.1	1.1	3.8	0.1	14.4	0.1	4.1	0.1	1.6	0.1	3.3	0.1	0.50	0.06	2.2	0.1	17.9	0.5	0.44	0.03	1.5	0.1	0.27	0.02	1.3	0.1	0.25	0.04
**22**	15.0	0.2	28.7	0.8	3.6	0.2	13.6	0.7	3.2	1.0	1.1	0.5	2.7	0.6	0.42	0.07	2.0	0.2	14.7	0.7	0.38	0.01	1.2	0.1	0.20	0.03	1.2	0.2	0.20	0.04
**23**	19.6	1.1	37.7	1.8	4.4	0.2	16.5	0.5	4.2	0.3	1.9	0.2	3.3	0.2	0.49	0.02	2.1	0.2	17.2	0.9	0.45	0.03	1.3	0.1	0.24	0.03	1.3	0.1	0.23	0.04
**24**	20.2	0.5	38.2	1.7	4.5	0.2	16.7	1.1	4.4	0.3	1.3	0.1	3.4	0.2	0.52	0.02	2.3	0.1	18.4	1.2	0.45	0.04	1.6	0.1	0.26	0.01	1.7	0.4	0.31	0.05
**25**	25.0	2.4	49.2	4.4	5.8	0.6	22.2	2.6	5.3	0.6	2.0	0.2	4.4	0.6	0.67	0.01	2.9	0.3	27.1	1.9	0.58	0.05	1.9	0.2	0.34	0.06	1.4	0.2	0.27	0.03
**26**	20.4	2.1	40.7	5.0	4.8	0.6	18.8	2.3	4.6	0.5	1.5	0.2	3.5	0.4	0.53	0.03	2.5	0.2	21.2	2.1	0.50	0.05	1.6	0.3	0.26	0.05	1.5	0.2	0.32	0.03
**27**	19.7	1.1	36.7	2.5	4.5	0.3	17.3	0.6	4.8	0.3	2.7	0.4	3.8	0.2	0.57	0.06	2.5	0.2	20.1	2.0	0.53	0.05	2.0	0.3	0.33	0.03	1.3	0.1	0.23	0.02
**28**	15.4	0.2	29.3	0.4	3.4	0.1	13.0	0.3	3.3	0.2	1.3	0.1	2.8	0.1	0.44	0.01	1.9	0.1	14.6	0.1	0.38	0.01	1.4	0.1	0.27	0.01	1.4	0.2	0.31	0.02
**29**	21.9	1.9	41.7	3.6	4.9	0.4	18.4	1.8	4.7	0.3	2.5	0.2	3.9	0.2	0.63	0.06	2.5	0.1	20.9	1.8	0.51	0.05	1.9	0.1	0.30	0.05	1.4	0.2	0.31	0.01
**30**	25.0	0.8	46.9	1.3	5.5	0.1	21.0	0.6	4.9	0.6	2.9	0.1	4.2	0.1	0.65	0.02	2.8	0.1	25.0	0.3	0.55	0.02	1.9	0.1	0.30	0.03	1.5	0.1	0.26	0.04
**31**	22.4	0.6	40.9	1.1	5.0	0.2	18.3	0.5	4.7	0.3	2.4	0.1	3.9	0.2	0.58	0.01	2.6	0.1	19.7	0.9	0.52	0.02	1.8	0.1	0.31	0.03	1.4	0.1	0.31	0.03
**32**	29.1	1.1	54.7	1.2	6.4	0.1	23.7	0.2	5.7	0.2	2.3	0.1	5.5	0.2	0.74	0.02	3.1	0.1	23.4	0.7	0.77	0.04	1.9	0.1	0.35	0.02	1.2	0.1	0.27	0.01
**33**	21.5	0.8	34.9	1.3	4.6	0.2	17.6	0.8	4.1	0.1	3.0	0.4	4.6	0.2	0.55	0.05	2.5	0.2	17.4	0.6	0.53	0.02	1.7	0.2	0.30	0.05	1.2	0.1	0.24	0.02
**34**	24.9	0.7	48.1	0.7	5.3	0.1	20.4	0.5	4.8	0.2	2.6	0.1	4.7	0.1	0.65	0.03	2.8	0.2	21.8	1.5	0.58	0.05	1.8	0.1	0.31	0.04	1.9	0.1	0.36	0.03
**35**	31.4	1.3	58.8	5.2	7.2	0.2	27.3	1.1	6.3	0.4	2.3	0.2	7.2	0.3	0.89	0.06	3.8	0.2	32.0	1.6	0.78	0.03	2.3	0.3	0.37	0.02	1.4	0.1	0.23	0.01
**36**	19.8	1.7	37.3	3.3	4.6	0.3	18.8	1.8	4.6	0.2	2.2	0.3	5.4	0.4	0.62	0.03	2.7	0.3	18.1	1.8	0.56	0.08	1.7	0.1	0.33	0.04	2.3	0.4	0.44	0.09
**37**	50.5	3.0	100.3	6.7	11.7	1.1	44.0	5.3	9.5	0.5	3.4	0.5	10.0	3.4	1.22	0.20	5.3	0.4	46.6	2.3	1.01	0.14	3.1	0.3	0.44	0.09	1.6	0.1	0.31	0.04
**38**	21.0	1.4	37.5	4.7	4.8	0.4	18.0	1.1	4.4	0.2	2.8	0.1	4.7	0.3	0.59	0.02	2.6	0.2	19.6	1.5	0.56	0.04	1.8	0.1	0.36	0.03	1.0	0.1	0.20	0.01
**Sign.**	**		**		**		**		**		**		**		**		**		**		**		**		**		**		**	

## Data Availability

Data is contained within the article and supplementary material.

## Supplementary Materials

The following supporting information can be downloaded at: https://www.mdpi.com/article/10.3390/plants11192579/s1, Table S1 Microwave programs for soil and CRM Soil (a) and olive and CRM Leaves (b); Table S2 Average amounts, standard deviations and recover percentage of REE in the INCT-OBTL-5 and 2586 CRM samples (in bold the certified values the others are informative ones). LOQ, in nanomoles Kg^−1^, and R^2^ from calibration curve. Table S3 Average amounts in micromoles Kg^−1^ and standard deviations of REE in Campo Carboj soil samples. Figure S1. UCC-normalised REE pattern of all Campo Carboj olive samples
