# Supplementary material for: The Pattern of Rare Earth Elements Like a Possible Helpful Tool in Traceability and Geographical Characterization of the Soil-Olive System (Olea europaea L.)
Source: Plants (Basel). 2022 Sep 30;11(19):2579. doi: 10.3390/plants11192579 (PMC9571631; doi:10.3390/plants11192579)

Table S1. Microwave programs for soil and CRM Soil (a) and olive and CRM Leaves (b)

(a)

Power	1600 Watt
Temperature Ramp	10 min (from R.T. to 200°C)
Working Temperature	200 °C
Working Time (at 200°C)	50 min

(b)

First step (Open vessel + HNO_3)	
Working Time	60 min
Second step (Closed vessel in microwave + H_2O_2)	
Power	1600 Watt
Temperature Ramp	10 min (from R.T. to 200°C)
Working Temperature	200 °C
Working Time (at 200°C)	50 min

Table S2. Average amounts, standard deviations and recover percentage of REE in the INCT-OBTL-5 and 2586 **CRM samples (in bold the certified values the others are informative ones). LOQ, in nanomoles Kg^{-1}, and R^2 from calibration curve.**

	INCT-OBTL-5 nanomoli/kg					SRM 2586 micromoli/kg						
	**C_{av}**	**σ**	**RSD%**	**CRM**	**REC%**	**C_{av}**	**σ**	**RSD%**	**CRM**	**REC%**	**LOQ**	**R^2**
Y	11888.4	314.9	2.6	10831.2	110	**234.2**	4.9	2.1	**230.6**	101.5	0.85	0.99998
**La**	**11785.5**	**496.8**	**4.2**	**12167**	**97**	**193.5**	5.8	3.0	**194.4**	**99.5**	1.23	0.99996
**Ce**	**20274.8**	**1054.3**	**5.2**	**21341.9**	**95**	**409.4**	18.4	4.5	**407.6**	**100.4**	1.54	0.99999
Pr	2384.7	106.5	4.4	2278.2	105	**47.3**	1.4	2.9	**48.1**	98.3	0.3	0.99998
**Nd**	**8918.2**	**339.8**	**3.8**	**9223.3**	**97**	**188.9**	2.8	1.5	**188.6**	**100.1**	0.62	0.99997
**Sm**	**1702.1**	**46.5**	**2.8**	**1755.3**	**97**	**41.3**	0.70	1.7	**40.0**	**103.1**	0.22	0.99997
**Eu**	**415.9**	**6.2**	**1.5**	**396.1**	**105**	**8.1**	0.15	1.8	**8.8**	**92.0**	0.18	0.99996
Gd	1699.3	37.4	2.2	1544.8	110	**37.5**	0.34	0.9	**38.4**	97.6	0.22	0.9928
**Tb**	**212.7**	**2.5**	**1.2**	**218.4**	**97**	**4.3**	0.03	0.8	**5.0**	**84.4**	0.08	0.99998
Dy	1157.5	13.5	1.2	1132.3	102	**27.9**	0.25	0.9	**28.9**	96.6	0.21	0.99999
Ho	217.7	3	1.4	209.2	104	6.1	0.07	1.2	6.7	90.6	0.14	0.99997
**Er**	**609.7**	**12**	**1.7**	**603.7**	**101**	19.9	0.26	1.3	19.7	**100.9**	0.16	0.99998
Tm	78.7	1.8	1.9	80.5	98	2.5	0.04	1.5	3.0	81.6	0.09	0.99998
**Yb**	**647.4**	**28.9**	**4.8**	**664.7**	**97**	**10.9**	0.23	2.1	**11.9**	**90.8**	0.14	0.99999
Lu	71.6	1.6	2.3	95.4	75	2.3	0.03	1.1	*	*	0.15	0.99999

Table S3. Average amounts in micromoles Kg^{-1} and standard deviations of REE in Campo Carboj soil samples.

	Pseudo-total		Bioavailability (DTPA)	
	average	± std. dev.	average	± std. dev.
La	142.2	17.0	15.2	4.6
Ce	314.9	27.9	26.6	3.4
Pr	33.5	4.1	4.1	1.2
Nd	129.3	16.9	17.4	5.0
Sm	24.1	3.0	3.6	1.0
Eu	5.0	0.7	0.8	0.2
Gd	23.9	3.1	3.8	1.0
Tb	2.9	0.4	0.5	0.1
Dy	13.6	1.9	2.5	0.6
Y	128.8	19.5	24.8	6.2
Ho	2.5	0.3	0.4	0.1
Er	6.7	1.0	1.1	0.2
Tm	0.8	0.1	0.14	0.03
Yb	5.0	0.7	0.8	0.2
Lu	0.7	0.1	0.12	0.02

Figure S1. UCC-normalised REE pattern of all Campo Carboj olive samples

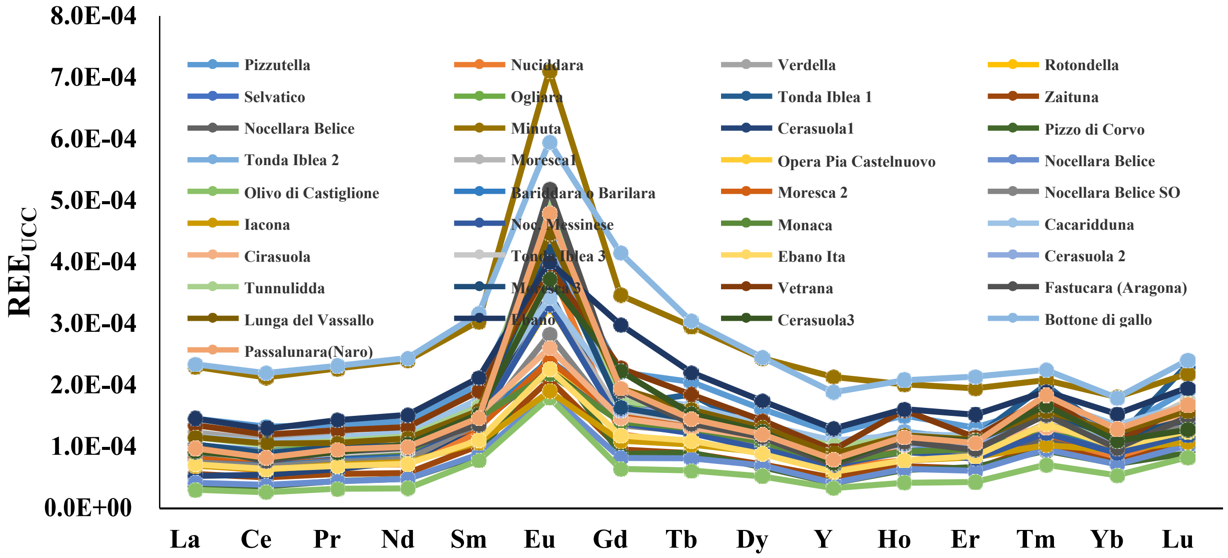

Supplement: Supplementary file 1 [file plants-11-02579-s001.zip › plants-1938508-supplementary (1).pdf]
